# Supplementary material for: Physiological changes in retinal layers thicknesses measured with swept source optical coherence tomography
Source: PLoS One. 2020 Oct 14;15(10):e0240441. doi: 10.1371/journal.pone.0240441 (PMC7556480; doi:10.1371/journal.pone.0240441)
Supplement: Attachment — Submitted filename: Responses to reviewers.docx [file pone.0240441.s002.docx]

Jun 03, 2020

Dear Editor-in-Chief,

Please find enclose the manuscript entitled “Physiological changes in retinal layers thicknesses measured with swept source optical coherence tomography (PONE-D-20-02557)”. We have improved the paper in response to your suggestions, and we would like it to be considered for publication in the *Plos One*.

As the corresponding author I confirm that all authors have read the revision and agree with changes.

We thank reviewers for providing us with very pertinent and helpful comments.

**RESPONSES TO REVIEWER 1**
Reviewer #1: The authors collects SS-OCT scans and analyzed the results from a large cohort which is interesting. However, the paper did not provide novel information of intraretinal layers over aging

1. The authors did not present the results of all intraretinal layers such as INL, OPL, ONL and PR. However, some selected layers and their combinations are presented and the majority of these presented layers are not commonly used in clinic. For example, we all use RNFL and GCIPL in addition to the retinal thickness.

*Response: Wide 3D+5LineCross protocol scan and Disc5.2x5.2-Superpixelgrid-200 export analysis don’t provide INL, OPL, ONL and PR since is focused in optic nerve and surrounding areas. Now a days Topcon company is improving their segmentation analysis.*

2. Although the SS-OCT scans a large area which may be better than the 6 x 6 mm scan area commonly used in clinic and research. On the other hand, the large area may prevent further comparison if the normality data is not available.

*Response: By the moment normality database for Triton is not available, as we know Topcon is working on it.*

3. The authors did not provide the details of scan settings such scan lines and area, and did not provide the details of 3D segmentation methods.

*Response: area is specified in materials and methods in line 135:*

- *”(12.0*9.0mm + 9.00mm overlap 8)”.*

*Number of scans have been added in line 136:*

*“We performed the 3D+5LineCross protocol (12.0*9.0mm + 9.00mm overlap 8), which allows 100.000 A scans/sec*

*Segmentation method has been clarified in line 154-156:*

*“Automated built-in calibration software, Topcon Advances Boundary Software (TABS) determined the distance between the delimiting lines in retina and choroidal plexus, establishing 7 boundaries and five layers.”*

4. The authors will need to provide a cross-sectional OCT image and show the segmentation boundaries and 3D map.

*Response: We have added the new image, which is now figure 1, so previous figure 1 is now figure 2, figure 2 is now figure 3 and figure 3 is now figure 4.*

*-In line (140-142):*

*“Figure 1: A: Location of 3D+5line cross OCT scans on retina. B: 26*26 grid centered on optic disc, the center area is not shown because the OCT thickness is always zero. C: cross-sectional OCT image and segmentation boundaries.”*

*Also, if reviewer consider there is a 3D diagram video that we can include.*

5. The age grouping is highly arbitrary and the authors need to provide the justification of the cut-off setting. It would be a good approach to use scatterplot to show the trend.

*Response: As we want to include elderly subjects and the youngest’s subjects are 20 years old, the difference between the eldest’s and the youngest was 60 years. Also, we were looking for a reasonable number of groups, and authors agreed that 4 was most appropriate, so the difference between groups is exactly 15 years.*

- *Data has been clarified in line 127:*

*“that were classified in four groups in steps of 15 years, as the difference between the youngest and the oldest is 60 years:”*

*Authors agree that the scatterplot graphic is not the appropriated one based on our results since the thickening and subsequent thinning will not be appreciated, just a progressive and linear thinning will be observed.*

5. The changes of intraretinal layers have been well documented and this manuscript did not report new findings, nor new database (since the device is not commonly used in the clinic).

*Response: Per our knowledge this is the first study that analyses retinal changes with age with SS-OCT and shows an 3D evolutive diagram for age and layers and mention the retinal thickening around the third decade of life. Also, the data analysis is novel and bigger than others, as we used a 26*26 grid to analyse quadrants and halves individually, not the traditional analysis based on ETDRS grid.*

6. The numbers appear to be wrong since the retinal thickness cannot be about 500,00 microns. It should be about 500.00 microns. Similarily, P should be < 0.001, instead of P < 0,001.

*Response: Table 1 and Table2 have been corrected.*

*Table 1: Mean ± standard deviation of four quadrants and halves for age groups in the full layers, retina and choroid measurement, and comparison of thickness between age groups. The asterisks show significant levels that overcome Bonferroni correction for multiple comparisons. Abbreviations: TS, temporal superior; TI, temporal inferior; NS, nasal superior; NI, nasal inferior.*

|  |  | Group 1  (20-34 years) | Group 2  (35-49 years) | Group 3  (50-64 years) | Group 4  (65-79 years) | P |
| --- | --- | --- | --- | --- | --- | --- |
| Full layers | TS Quadrant | 498.67±40.41 | 517.52±71.87 | 485.86±79.41 | 458.84±52.42 | <0.001* |
|  | TI Quadrant | 470.53±50.57 | 493.19±73.98 | 463.30±78.88 | 418.36±47.21 | <0.001* |
|  | NS Quadrant | 479.50±43.65 | 480.96±68.32 | 465.49±89.01 | 426.43±54.78 | <0.001* |
|  | NI Quadrant | 436.80±35.97 | 449.07±68.93 | 427.20±82.69 | 389.57±45.18 | <0.001* |
|  | Superior Half | 489.09±39.43 | 499.24±68.04 | 475.68±83.03 | 442.63±51.55 | <0.001* |
|  | Inferior Half | 453.67±40.77 | 471.13±69.78 | 445.25±79.36 | 403.97±44.68 | <0.001* |
|  | Nasal Half | 458.15±38.27 | 465.02±67.74 | 446.35±85.07 | 408.00±47.52 | <0.001* |
|  | Temporal Half | 484.60±44.81 | 505.35±71.76 | 474.58±78.35 | 438.60±47.09 | <0.001* |
| Retina | TS Quadrant | 300.15±9.62 | 303.03±13.05 | 294.89±14.05 | 290.65±10.68 | <0.001* |
|  | TI Quadrant | 293.30±10.88 | 298.22±14.59 | 294.78±15.79 | 285.90±13.29 | <0.001* |
|  | NS Quadrant | 274.31±12.44 | 272.71±14.01 | 266.56±18.82 | 262.47±13.48 | <0.001* |
|  | NI Quadrant | 259.40±8.85 | 261.13±10.94 | 255.70±16.01 | 255.04±16.13 | 0.012 |
|  | Superior Half | 287.23±9.01 | 287.87±12.24 | 280.73±15.29 | 276.56±11.16 | <0.001* |
|  | Inferior Half | 277.85±8.47 | 279.68±12.06 | 275.24±14.68 | 270.47±13.13 | <0.001* |
|  | Nasal Half | 266.85±9.31 | 266.92±11.81 | 261.13±16.75 | 258.76±14.06 | <0.001* |
|  | Temporal Half | 298.22±9.38 | 300.63±12.80 | 294.84±14.42 | 288.27±11.30 | <0.001* |
| Choroid | TS Quadrant | 173.28±40.68 | 203.81±77.25 | 168.16±74.45 | 145.42±48.17 | <0.001* |
|  | TI Quadrant | 149.75±46.90 | 179.46±72.31 | 145.66±73.86 | 109.60±39.44 | <0.001* |
|  | NS Quadrant | 175.66±43.38 | 193.17±68.84 | 174.84±82.28 | 140.19±47.74 | <0.001* |
|  | NI Quadrant | 147.86±34.92 | 169.42±68.43 | 147.11±77.33 | 110.51±35.24 | <0.001* |
|  | Superior Half | 174.47±39.34 | 198.49±71.18 | 146.39±77.27 | 142.81±45.52 | <0.001* |
|  | Inferior Half | 148.80±38.36 | 174.44±68.99 | 146.39±74.44 | 110.05±35.90 | <0.001* |
|  | Nasal Half | 161.76±38.04 | 181.30±67.82 | 160.98±78.97 | 125.35±38.98 | <0.001* |
|  | Temporal Half | 161.52±43.20 | 191.64±73.73 | 156.91±73.27 | 127.51±41.66 | <0.001* |

*Table 2: Mean ± standard deviation of four quadrants and halves for age groups in the retinal nerve fiber layer, GCL+ and GCL++, and comparison of thickness between age groups. The asterisks show significant levels that overcome Bonferroni correction for multiple comparisons, Abbreviations: TS, temporal superior; TI, temporal inferior; NS, nasal superior; NI, nasal inferior.*

|  |  | Group 1  (20-34 years) | Group 2  (35-49 years) | Group 3  (50-64 years) | Group 4  (65-79 years) | P |
| --- | --- | --- | --- | --- | --- | --- |
| RNFL | TS Quadrant | 97.10±10.17 | 103.01±10.05 | 97.53±11.79 | 96.36±11.70 | 0.001* |
|  | TI Quadrant | 93.16±11.55 | 97.63±9.57 | 97.12±13.62 | 90.49±12.03 | <0.001* |
|  | NS Quadrant | 88.89±12.20 | 88.70±10.66 | 87.13±14.22 | 85.19±12.04 | 0.222 |
|  | NI Quadrant | 77.72±10.44 | 82.00±7.50 | 80.94±11.39 | 84.49±15.81 | 0.003* |
|  | Superior Half | 92.99±6.39 | 95.87±8.03 | 92.33±10.55 | 90.78±9.94 | 0.009 |
|  | Inferior Half | 85.44±6.15 | 89.81±6.08 | 89.03±9.17 | 97.49±10.30 | 0.007* |
|  | Nasal Half | 83.30±9.31 | 85.35±8.02 | 84.03±11.82 | 84.84±13.16 | 0.687 |
|  | Temporal Half | 95.13±9.60 | 100.32±7.41 | 97.33±11.89 | 93.42±10.73 | <0.001* |
| GCL + | TS Quadrant | 48.87±4.26 | 47.70±5.29 | 47.01±4.69 | 45.10±4.35 | <0.001* |
|  | TI Quadrant | 53.07±6.21 | 52.36±6.07 | 51.11±5.51 | 49.70±5.63 | 0.001* |
|  | NS Quadrant | 40.42±2.99 | 39.27±3.22 | 39.44±4.31 | 38.40±3.82 | 0.011 |
|  | NI Quadrant | 41.06±3.84 | 39.69±3.67 | 39.13±3.93 | 37.79±5.42 | <0.001* |
|  | Superior Half | 44.65±3.05 | 43.49±4.3 | 43.23±3.76 | 41.75±3.38 | <0.001* |
|  | Inferior Half | 47.06±4.14 | 46.03±4.14 | 45.15±3.85 | 43.75±4.53 | <0.001* |
|  | Nasal Half | 40.47±3.15 | 38.48±3.17 | 39.29±3.72 | 38.09±3.87 | <0.001* |
|  | Temporal Half | 50.97±4.82 | 50.03±5.44 | 49.06±4.74 | 47.40±4.56 | <0.001* |
| GCL++ | TS Quadrant | 145.98±9.61 | 150.72±11.23 | 144.55±11.72 | 141.47±13.00 | <0.001* |
|  | TI Quadrant | 146.23±9.25 | 150.01±10.43 | 148.25±13.14 | 140.20±13.29 | <0.001* |
|  | NS Quadrant | 129.28±13.08 | 128.00±11.89 | 126.49±15.07 | 123.45±13.76 | 0.046 |
|  | NI Quadrant | 118.78±10.08 | 121.70±8.98 | 120.01±11.65 | 122.10±16.72 | 0.263 |
|  | Superior Half | 137.63±8.03 | 139.36±10.03 | 135.52±11.74 | 132.46±12.03 | 0.001* |
|  | Inferior Half | 132.51±6.73 | 135.85±8.45 | 134.13±10.16 | 131.15±12.68 | 0.015 |
|  | Nasal Half | 124.03±10.07 | 124.85±9.62 | 123.25±12.53 | 122.78±14.55 | 0.709 |
|  | Temporal Half | 146.10±8.40 | 150.36±9.29 | 146.40±11.79 | 140.83±12.39 | <0.001* |

7. The simple conclusion is the thinning of retinal layer, which is well documented. However, the authors did not provide the thinning rate.

*Response: Thinning rate has been calculated and added*

- *In results (line 269-274):*

*“Except the GCL+ with a linear thinning ratio of -0.050 mm/year, the tendency in the other layers differs. A thickening ratio of 0.476 mm/year for the full layers complex, 0.010 mm/year for the retina, 0.856 mm/year for the choroid, 0.125 mm/years for the RNFL and 0.087 mm/years for the GCL++ is observed until de third decade of life. Subsequently a thinning ratio of -1.406 mm/year for the full layers complex, -0.058 mm/year for the retina, -1.364 mm/year for the choroid, -0.084 mm/years for the RNFL and -0.131 mm/years for the GCL++ is observed from de third decade of life.”*

8. There are missing data in the center as shown in Fig. 3. Why is the center area trimmed and shown black. Did the center of the fovea was aligned from each map? Or this is just a numerical demonstration.

*Response: numerical demonstration is centered on optic disc (trimmed area). Mean and SD statistical analysis por each age group is calculated separately and then Abacus process numerical data in images.* *Figure 3 shows the mean thickness for each group for the different analyzed layers. The thickness value is extracted from the OCT protocol. Therefore, the OCT thickness value of each box of the grid, is extracted and then ploted in a contour map.* *The center area is always shown in black because the OCT thickness is always zero. Besides, the center of the fovea is always aligned.*

**RESPONSES TO REVIEWER 2**

Reviewer #2: a thickening until de third decade of live,: I will change it to third decade of life not live.

*Response: corrected in line 41*

line 75: possible not possible

*Response: corrected in line 74*

line 84: systemic conditions like diabetes mellitus 11 or migraine: why considering migraine systemic condition?

*Response: information has been corrected and clarified.*

- *In introduction (line 83-84):*

*“pathologies with unclear physiopathology such as migraine”*

Line 91: advent: I will write it advantage instead

*Response: corrected in line 93*

line 93: Even so,: should be even though

*Response: corrected in line 94*

did U mention in this article the race of the patients, like white, AA or east Asian...etc, I mean U did not mention the ethnicity of your study population

*Response: information has been corrected and clarified.*

- *In materials and methods (line 108):*

*“Between 2015 and 2018, 480 healthy withe caucasian patients were recruited for this cross-sectional study”*

**ADDITIONAL REQUIREMENTS**

1. Please ensure that your manuscript meets PLOS ONE's style requirements, including those for file naming. The PLOS ONE style templates can be found at https://journals.plos.org/plosone/s/file?id=wjVg/PLOSOne_formatting_sample_main_body.pdf and https://journals.plos.org/plosone/s/file?id=ba62/PLOSOne_formatting_sample_title_authors_affiliations.pdf

*Response: Titles, bibliography, references, tables and figures have been adapted to Plos One guidelines.*

2. Thank you for including your ethics statement:  "All subjects gave detailed consent to participate in this study, which was conducted in accordance with the guidelines determined by the Ethics Committee of the Miguel Servet Hospital and the principles of the Declaration of Helsinki."

Please amend your current ethics statement to confirm that your named institutional review board or ethics committee specifically approved this study.

Once you have amended this/these statement(s) in the Methods section of the manuscript, please add the same text to the “Ethics Statement” field of the submission form (via “Edit Submission”).

*Response: Data has been amended in line 120:*

- *The written informed consent for participants and for the study protocol was approved by the Ethics Committee of Clinic Research in Aragon (CEICA) and by the Ethics Committee of Miguel Servet University Hospital, in Zaragoza, Spain, which specifically approved the study procedures*

For additional information about PLOS ONE ethical requirements for human subjects research, please refer to http://journals.plos.org/plosone/s/submission-guidelines#loc-human-subjects-research.

3. Please include a copy of Table 4 which you refer to in your text on page 9.

*Response: Mistake has been corrected in line 220 as is not table 4, is table 2.*

We have submitted the response to reviewers and a new manuscript with changes and corrections.

Thank you very much for improving our paper.

E. Garcia-Martin
